# Gigantic-oxidative atomic-layer-by-layer epitaxy for artificially designed complex oxides

**DOI:** 10.1093/nsr/nwae429

**Published:** 2024-11-27

**Authors:** Guangdi Zhou, Haoliang Huang, Fengzhe Wang, Heng Wang, Qishuo Yang, Zihao Nie, Wei Lv, Cui Ding, Yueying Li, Jiayi Lin, Changming Yue, Danfeng Li, Yujie Sun, Junhao Lin, Guang-Ming Zhang, Qi-Kun Xue, Zhuoyu Chen

**Affiliations:** Department of Physics and Guangdong Basic Research Center of Excellence for Quantum Science, Southern University of Science and Technology, Shenzhen 518055, China; Department of Physics and Guangdong Basic Research Center of Excellence for Quantum Science, Southern University of Science and Technology, Shenzhen 518055, China; Quantum Science Center of Guangdong-Hong Kong-Macao Greater Bay Area, Shenzhen 518045, China; Department of Physics and Guangdong Basic Research Center of Excellence for Quantum Science, Southern University of Science and Technology, Shenzhen 518055, China; Department of Physics and Guangdong Basic Research Center of Excellence for Quantum Science, Southern University of Science and Technology, Shenzhen 518055, China; Department of Physics and Guangdong Basic Research Center of Excellence for Quantum Science, Southern University of Science and Technology, Shenzhen 518055, China; Department of Physics and Guangdong Basic Research Center of Excellence for Quantum Science, Southern University of Science and Technology, Shenzhen 518055, China; Department of Physics and Guangdong Basic Research Center of Excellence for Quantum Science, Southern University of Science and Technology, Shenzhen 518055, China; Quantum Science Center of Guangdong-Hong Kong-Macao Greater Bay Area, Shenzhen 518045, China; Department of Physics and Guangdong Basic Research Center of Excellence for Quantum Science, Southern University of Science and Technology, Shenzhen 518055, China; Department of Physics and Guangdong Basic Research Center of Excellence for Quantum Science, Southern University of Science and Technology, Shenzhen 518055, China; Department of Physics, South China University of Technology, Guangzhou 510006, China; Department of Physics and Guangdong Basic Research Center of Excellence for Quantum Science, Southern University of Science and Technology, Shenzhen 518055, China; Quantum Science Center of Guangdong-Hong Kong-Macao Greater Bay Area, Shenzhen 518045, China; Department of Physics, City University of Hong Kong, Hong Kong, China; Department of Physics and Guangdong Basic Research Center of Excellence for Quantum Science, Southern University of Science and Technology, Shenzhen 518055, China; Quantum Science Center of Guangdong-Hong Kong-Macao Greater Bay Area, Shenzhen 518045, China; Department of Physics and Guangdong Basic Research Center of Excellence for Quantum Science, Southern University of Science and Technology, Shenzhen 518055, China; Quantum Science Center of Guangdong-Hong Kong-Macao Greater Bay Area, Shenzhen 518045, China; State Key Laboratory of Low-Dimensional Quantum Physics, Department of Physics, Tsinghua University, Beijing 100084, China; Frontier Science Center for Quantum Information, Beijing 100084, China; Department of Physics and Guangdong Basic Research Center of Excellence for Quantum Science, Southern University of Science and Technology, Shenzhen 518055, China; Quantum Science Center of Guangdong-Hong Kong-Macao Greater Bay Area, Shenzhen 518045, China; Department of Physics and Guangdong Basic Research Center of Excellence for Quantum Science, Southern University of Science and Technology, Shenzhen 518055, China; Quantum Science Center of Guangdong-Hong Kong-Macao Greater Bay Area, Shenzhen 518045, China

**Keywords:** oxide thin film, epitaxy, nickelate, cuprate, superconductors

## Abstract

In designing material functionalities for transition metal oxides, lattice structure and *d*-orbital occupancy are key determinants. However, the modulation of these two factors is inherently limited by the need to balance thermodynamic stability, growth kinetics and stoichiometry precision, particularly for metastable phases. We introduce a methodology, namely gigantic-oxidative atomic-layer-by-layer epitaxy (GOALL-Epitaxy), to enhance oxidation power by three to four orders of magnitude beyond conventional pulsed laser deposition and oxide molecular beam epitaxy, while ensuring atomic-layer-by-layer growth of the designed complex structures. Thermodynamic stability is markedly augmented with stronger oxidation at elevated temperatures, whereas growth kinetics is sustained by using laser ablation at lower temperatures. We demonstrate the accurate growth of complex nickelates and cuprates—especially an artificially designed structure with alternating single and double NiO_2_ layers that possess distinct nominal d-orbital occupancy, as a parent of the high-temperature superconductor. GOALL-Epitaxy enables material discovery within the vastly broadened growth parameter space.

## Teaser

The gigantic-oxidative atomic-layer-by-layer epitaxy (GOALL-Epitaxy) method substantially augments the oxidation power by orders of magnitude, enabling atomically precise construction of artificially designed metastable complex oxide structures.

## INTRODUCTION

In transition metal oxides, complex interplays among charge, spin, orbital and lattice degrees of freedom give rise to a rich spectrum of phenomena such as metal–insulator transitions, magnetism, ferroelectricity and superconductivity [1,2]. These intertwined orders are rooted in the delicate balance of similar, yet competing and correlated, energy scales [3]. For instance, the transition from metal to antiferromagnetic insulator depends on the comparison between the *d*-orbital bandwidth and the *d*–*d* Coulomb interaction. Further complexity arises if the oxygen ligand-to-metal charge-transfer energy falls below the Coulomb interaction, shifting the determination to a finer energy-scale comparison [4]. This nuanced energy landscape underscores the diverse manifestations of physical properties in transition metal oxides, setting the stage for the design of correlated electron systems.

In designing complex oxides for desired functionalities, two important factors of consideration are the lattice structure and the *d*-orbital occupancy of the transition metal ions. The lattice structure not only determines the dimensionality of the active functional layer (e.g. 3D versus 2D), but also governs interfacial coupling between layers within the designed structures [5–9]. Simultaneously, the number of correlated electrons that reside in the *d* orbitals at each lattice site directly links to the electronic structure and the Fermi level [10–12]. However, the interplay between the lattice structure and the *d*-orbital occupancy is complex and often interdependent [13,14]. Perturbations of the lattice structures, such as oxygen octahedra rotation [7,15,16] or Jahn–Teller distortion [17], are closely related to the electron count in *d* orbitals due to crystal field anisotropy, illustrating the intricate relationship between these fundamental parameters.

The successful growth of designed complex oxide systems hinges on two critical abilities: (i) the independent control over the intertwined factors of lattice structure and transition metal *d*-orbital occupancy and (ii) the stabilization and manipulation of metastable phases. The achievement of these objectives necessitates careful attention to thermodynamic stability, growth kinetics, stoichiometry precision and the ability to accurately control oxygen content *in situ* over wide ranges. Molecular beam epitaxy (OMBE) and pulsed laser deposition (PLD) stand out as premier techniques for the crafting of complex oxide epitaxial single-crystalline thin films and heterostructures [18,19]. While these thin-film techniques facilitate the creation and exploration of artificial structures and complex physical phenomena, their effectiveness is somewhat diminished for phases that demand substantial oxidation compared with methods such as high-pressure synthesis [20,21]. OMBE—especially when alternately shuttered element sources are employed—offers meticulous control over cation stoichiometry and supports atomic-layer-by-layer growth [22–24], whereas PLD is prized for its simplicity, versatility of materials and capability for higher-pressure environments to enhance thermodynamic stability [25,26]. Nonetheless, each technique presents challenges: OMBE is limited by the vapor pressure of the elements and requires a low-pressure environment for the transport of evaporated materials to the substrate, thus constraining its oxidation potential; PLD can lead to stoichiometric imbalances and is less effective for materials with complex and large unit cells (UC), such as Ruddlesden–Popper (RP) phases. To address these comprehensive requirements, we present the gigantic-oxidative atomic-layer-by-layer epitaxy (GOALL-Epitaxy) method, detailed subsequently.

## RESULTS

**Figure 1.**
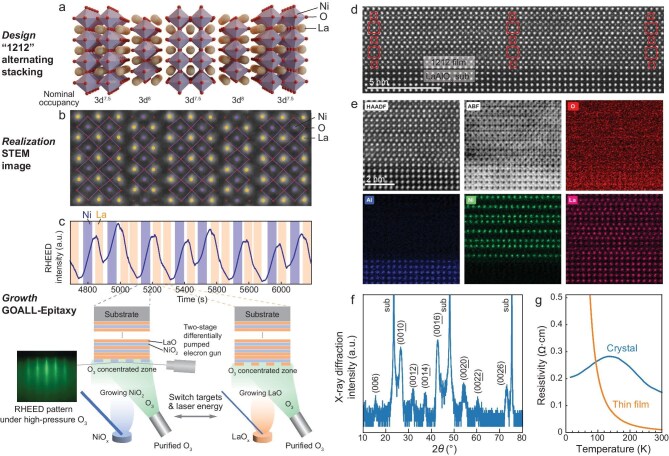
Growth of a designed complex structure with gigantic-oxidative atomic-layer-by-layer epitaxy (GOALL-Epitaxy). (a) Schematic of a designed complex structure, featuring alternating stacking of single and double layers of NiO_2_ (denoted as ‘1212’). This structure does not belong to the series of the Ruddlesden–Popper phase. (b) Magnified scanning transmission electron microscopy (STEM) image of the grown film, showing a region of the lattice structure that is the same as that depicted in (a). Atom positions are determined based on both high-angle annular dark field (HAADF) and annular bright field (ABF) images. (c) Reflective high-energy electron diffraction (RHEED) intensity oscillations as a function of time. Shaded backgrounds represent the durations of NiO*_x_* and LaO*_x_* targets being ablated. Lower schematics describe how a complex structure is constructed in an atomic-layer-by-layer way in the GOALL-Epitaxy set-up. (d) Larger-field-of-view HAADF image of the atomically sharp 1212 film and LaAlO_3_ substrate interface. Squares are visual guides for the single-NiO_2_ and double-NiO_2_ structures. (e) HAADF, ABF and atomically resolved energy-dispersive X-ray spectroscopy (EDS, for O, Al, Ni and La, respectively) images of the same region of the lattice. Alternating single and double layers of NiO_2_ are exhibited. (f) X-ray diffraction (XRD) in log scale of a 10-nm 1212 film. (g) Resistivity as a function of temperature for a 20-nm 1212 film and a crystal.

Figure 1 illustrates the operational principles of GOALL-Epitaxy with a prominent example growth of an artificially designed nickelate structure as a parent for high-temperature superconductivity [27]. Consisting of La, Ni and O, this structure features the alternating stacking of single- and double-layer NiO_2_ planes (thus denoted ‘1212’), with nominal 3*d*^8^ and 3*d*^7.5^ occupancy, respectively (Fig. 1a). The 1212 structure can be regarded as a combination of La_2_NiO_4_ and La_3_Ni_2_O_7_ RP phases, the former of which is an antiferromagnetic insulator and the latter of which was recently found to host superconductivity at liquid-nitrogen temperatures under high pressure [21]. Thanks to the flexibility of GOALL-Epitaxy, the 1212 structure can be successfully realized in thin-film form, as shown by the scanning transmission electron microscopy (STEM) image with atom positions identified in Fig. 1b.

In general, upon receiving a task of growth, the designed structure is broken down into its constituent oxide atomic layers (LaO and NiO_2_, in this case, schematized in Fig. 1c). Each atomic layer is associated with a specific oxide target (i.e. LaO*_x_* and NiO*_x_*). After the optimal growth temperature and oxidation conditions have been set, atomic layers are successively deposited onto a chosen substrate through pulsed laser ablation of these oxide targets [18,28]. The number of pulses required for each complete layer, varying from a few tens to several hundreds, is determined by the properties of the target material and the selected laser energy. Layers are deposited in a programmed sequence that reflects the design, with the entire growth process monitored in real time by using reflective high-energy electron diffraction (RHEED), allowing the immediate characterization of elemental and layer completion information [29], facilitating the atomic-layer-by-layer growth mode [19,28,30–33]. In the example case of the 1212 structure, one RHEED oscillation cycle corresponds to one LaO–NiO_2_–LaO–NiO_2_–LaO (a double-NiO_2_ layer) block plus one LaO–NiO_2_–LaO (a single-NiO_2_ layer) block, realized by sequential depositing using LaO*_x_*, NiO*_x_*, LaO*_x_*, NiO*_x_*, LaO*_x_* and LaO*_x_*, NiO*_x_*, LaO*_x_* targets.

Expanding upon atomic-layer-by-layer growth, the GOALL-Epitaxy technique employs purified ozone with a specially designed ozone nozzle (with an inner diameter of 10 mm) aimed directly at the sample, positioned closely (∼4 cm) to establish a highly concentrated oxidation zone close to the substrate (see Fig. S1 for simulation analysis). This geometry amplifies the oxidation power by around half an order of magnitude—a crucial enhancement given the rapid decomposition of ozone molecules upon contact with the heated sample stage. The ozone gas is purified in a liquefaction unit positioned close to the ozone inlet of the chamber to minimize decomposition during transport. The RHEED is equipped with a two-stage differential pump system, enabling the atomic-layer-by-layer growth mode to sustain ozone chamber pressures of ≤0.1 mbar. In contrast to OMBE, GOALL-Epitaxy synergizes the high-energy plasma plume that is generated by laser pulses—capable of withstanding high chamber ozone pressures for effective material transport to the substrate. Under strong oxidative conditions, the theoretical stoichiometry of GOALL-Epitaxy is as precise as 0.1% and has been experimentally demonstrated to be no worse than 0.3% (see Fig. S2). Additionally, the chamber pressure can be tuned in accordance with the laser fluence to ensure that ablated particles reach the substrate surface with a suitable kinetic energy, causing neither excessive damage to the grown structure nor insufficient surface diffusion. In the example case of the 1212 structure, the oxidative environment was meticulously set to 4 × 10^−4^ mbar of ozone plus 9.6 × 10^−3^ mbar of oxygen a with laser fluence of 1.4 J/cm^2^ for both the LaO*_x_* and the NiO*_x_* targets. For nominally distinct valences of nickel in different phases, the growth in principle necessitates different oxidative strengths. The mixed oxygen–ozone condition is chosen to accommodate the growth of both single-layer and double-layer structures.

A STEM high-angle annular dark field (HAADF) image with a larger field of view (Fig. 1d) shows a coherent 1212 lattice structure with a sharp substrate/film interface. Atomically resolved energy-dispersive X-ray spectroscopy (EDS) images exhibit element-selective signals (Fig. 1e). Within the field of view shown and all other EDS images measured (not shown here), we did not observe obvious interdiffusion between Al and Ni atoms at the interface. On top of the atomically flat substrate, single and double layers of Ni are alternatively stacked, signifying the high-quality realization of the artificially designed complex oxide lattice. X-ray diffraction (XRD, Fig. 1f) displays nearly all predicted peaks within the measurable range, except for those overlapping with the substrate peaks and (0024) due to lower symmetry. Low-temperature resistivity measurement of our grown film exhibits pure insulating behavior in contrast to that of the bulk crystal [27], which features a down bending below 140 K.

**Figure 2.**
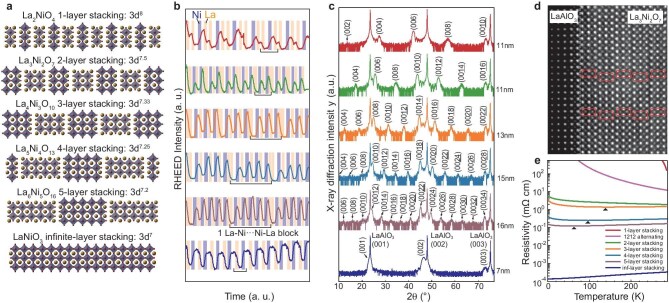
Growth of complex nickelate structures with *in situ* reduction. (a) Schematic structures of a series of Ruddlesden–Popper phases of nickelates. (b) RHEED oscillations corresponding to each of the designs in (a). Shaded backgrounds represent durations of NiO*_x_* and LaO*_x_* targets being ablated. (c) XRD corresponding to each of the thin films synthesized according to the design. Film thickness ranges from 10 to 20 nm. (d) STEM HAADF image of a grown double-layer stacking structure, La_3_Ni_2_O_7_. Rectangles are visual guides to highlight the LaO–NiO_2_–LaO–NiO_2_–LaO blocks as fundamental units that construct the structure. (e) Resistivity–temperature curves for varied synthesized films. Filled triangles indicate where the resistivity starts to increase with the temperature decrease.

Figure 2a–e illustrates the RP phases with varying numbers of transition metal oxide layers, using Ni as an example due to its high oxidation requirements. These variations correspond to different 3*d*-orbital occupancies (Fig. 2a). As carrier transport occurs within the NiO_2_ layers, the number of stacking layers controls the effective dimensionality of the electronic system: between adjacent blocks, the NiO_2_ layers are separated by two insulating LaO layers and the alignment of the oxygen octahedra in the lattice is shifted by approximately one bond length, reducing the probability of carrier hopping. The RHEED oscillations (Fig. 2b) reveal that different sequences of ablating La and Ni (i.e. LaO*_x_* and NiO*_x_* targets) yield various structural stackings, with the steady oscillation intensity indicating stoichiometric growth (example RHEED patterns shown in Fig. S3). The XRD spectrum for each structure (Fig. 2c) shows consecutive Bragg peaks along the out-of-plane axis, confirming periodic lattice structures. An STEM image of the two-layer structure of La_3_Ni_2_O_7_ (Fig. 2d) confirms the coherent growth on the LaAlO_3_ substrate and alternately positioned LaO–NiO_2_–LaO–NiO_2_–LaO blocks. Systematic resistivity–temperature curves for different layer stacking configurations (Fig. 2e) reveal that, whereas infinite-layer LaNiO_3_ displays metallic behavior, structures with five to two layers exhibit a metal–insulator transition at low temperatures, likely due to reduced dimensionality. The resistivity tends to increase as the number of layers decreases, largely as a result of changes in electron occupancy in the 3d orbitals, with the single-layer case (La_2_NiO_4_) being highly insulating, aligning with prior findings [32,33]. The enhancements in growth thermodynamics and kinetics provided by GOALL-Epitaxy enable the extension of the growth temperature range for LaNiO_3_. Specifically, the lower temperature limit is extended to 350°C under a chamber pressure of 2 × 10^−5^ mbar of O_3_, while the upper temperature limit reaches 900°C at a chamber pressure of 0.1 mbar of O_3_ (see Fig. S4). The quality of the LaNiO_3_ growth enables a wide tunable range of in-plane coherent strain that reaches ≤4.5% (see Fig. S5).

Upon growing the desired complex structures, for independent control of transition metal d-orbital occupancy over wide ranges while keeping structural coherence, we implement *in situ* reduction via atomic hydrogen in a dedicated reduction chamber [34–36], as illustrated in Fig. S6a. The thermally activated atomic hydrogen source is positioned vertically, ∼20 cm below the sample, to ensure a consistent flux (∼3 × 10^15^ atoms/cm^2^s) across the sample surface, which is important for the achievement of a spatially uniform reduction rate. The XRD data, as presented in Fig. S6b, demonstrate the capability to precisely adjust the oxygen content from (La,Sr)NiO_3_ with ∼3*d*^6.8^ configuration to (La, Sr)NiO_2_ corresponding to ∼3*d*^8.8^ by varying the atomic hydrogen flux and annealing temperature. Besides atomic hydrogen, reduction through the deposition of a thin reductant metal layer, such as evaporated Al from an effusion cell [37], is also possible in our reduction chamber.

**Figure 3.**
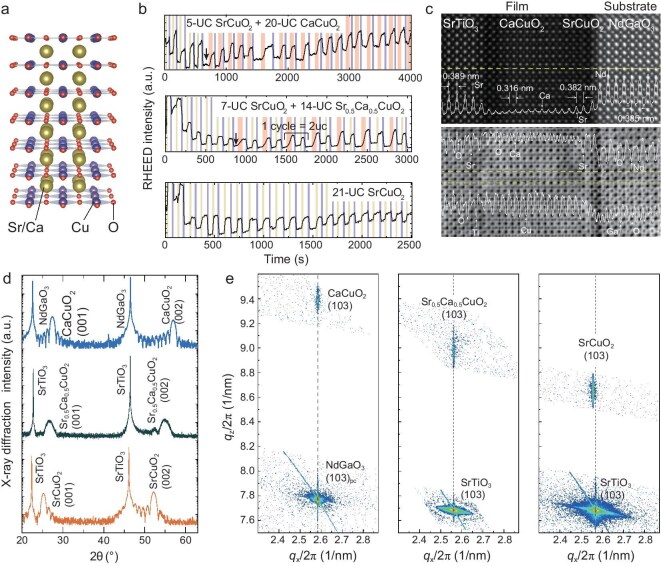
Growth of infinite-layer cuprates. (a) Schematic lattice structure of infinite-layer cuprates. (b) Example RHEED oscillations and patterns of CaCuO_2_ growth with SrCuO_2_ buffer on NdGaO_3_ substrate (top), Sr_0.5_Ca_0.5_CuO_2_ with SrCuO_2_ buffer and SrCuO_2_ growth on SrTiO_3_ substrates (middle and bottom). Blue, pink, and yellow shaded backgrounds represent the durations of the CuO*_x_*, SrO*_x_* and CaO*_x_* targets being ablated, respectively. Note the one cycle of the sequential depositions of CaO*_x_*, CuO*_x_*, SrO*_x_* and CuO*_x_*, corresponding to 2-UC Sr_0.5_Ca_0.5_CuO_2_. (c) STEM HAADF (top) and ABF (bottom) images of a SrTiO_3_/CaCuO_2_/SrCuO_2_/NdGaO_3_ sample, with the inset showing the image intensity as a function of the distance. (d and e) XRD spectra along the out-of-plane axis and reciprocal space mappings (RSM) of the three representative samples.

The next example showcases the growth of infinite-layer cuprate structures (Fig. 3a), with the maximized oxidation power of GOALL-Epitaxy. The infinite-layer structure represents the most fundamental parent of cuprate superconductors, characterized by the uninterrupted stacking of CuO_2_ planes, interspersed with alkaline earth ions [38,39]. Mastering the growth and manipulation of this structure is crucial for designing and creating new cuprate superconductors, although the production of high-quality crystals faces significant challenges due to their thermodynamic metastable nature [26,28]. Achievement of their growth necessitates exceptionally strong oxidation conditions: a more powerful oxidation environment promotes thermodynamic stability at elevated growth temperatures, thus enhancing growth kinetics and resulting in higher crystalline quality. In previous experiments in which OMBE and PLD were used, the growth temperature typically ranged between 550°C and 600°C due to limited oxidation capabilities [28,40–42]. By utilizing stronger oxidation, which is achievable by using GOALL-Epitaxy, the stable growth of infinite-layer cuprates can be achieved at temperatures up to 700°C, markedly exceeding those of earlier methods, indicating an enhanced thermodynamic stability. Figure 3b shows three example growth processes of CaCuO_2_ (with SrCuO_2_ buffer), Sr_0.5_Ca_0.5_CuO_2_ (with SrCuO_2_ buffer) and SrCuO_2_. Intriguingly, to achieve a 1 : 1 stoichiometry between the Sr and Ca in Sr_0.5_Ca_0.5_CuO_2_, we alternate the deposition of one atomic layer of Sr with one atomic layer of Ca; thus, one cycle of RHEED oscillation corresponds to the formation of two UC. In Fig. 3c, an STEM image demonstrates the coherent CaCuO_2_ thin film with a 20-UC SrTiO_3_ capping layer and a 3-UC SrCuO_2_ buffer layer grown on the NdGaO_3_ substrate. The atoms at the interface are clearly visible and consistent with the designed expectations. XRD data (Fig. 3d) reveal systematic variations in the out-of-plane lattice constant with different alkaline–earth element compositions. After-growth surface quality characterizations by using *in situ* RHEED and *ex situ* atomic force microscopy are shown in Figs S7 and S8. The slender reciprocal spots confirm the crystallinity of these cuprate thin films that were prepared by using GOALL-Epitaxy (Fig. 3e).

## DISCUSSION AND CONCLUSION

**Figure 4.**
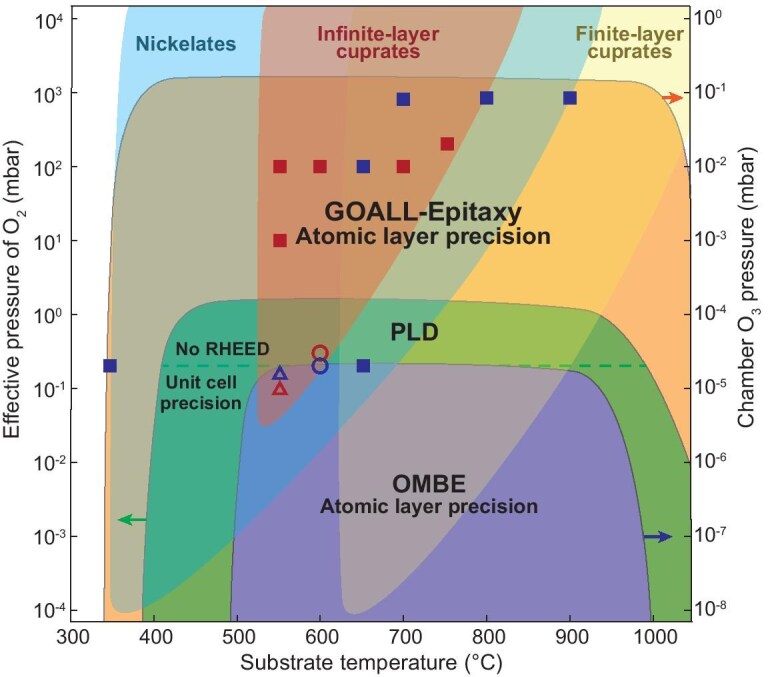
New frontier in the growth parameter space. The parameter spaces covered by the GOALL-Epitaxy, PLD and OMBE techniques are identified by different shaded regions delineated with solid lines, ranging from large to small, respectively. The parameter spaces where nickelates (here represented by ReNiO_3_, where Re = rare earth), infinite-layer cuprates and finite-layer cuprates (such as La_2_CuO_4_, YBa_2_Cu_3_O_7–δ_, etc.) can be grown are indicated from left to right by areas without border lines. For the PLD technique, the dashed line distinguishes the regions in which RHEED is applicable or not. Solid squares, empty circles and empty triangles represent the example growth parameters by using GOALL-Epitaxy, PLD [25,26,40] and OMBE [33,41–43], respectively. Blue and red symbols correspond to nickelates and infinite-layer cuprate growth, respectively.

Figure 4 summarizes the parameter space covered by various oxide thin-film techniques and the parameters required for different material systems. GOALL-Epitaxy exhibits oxidation power that significantly surpasses that of conventional PLD by three orders of magnitude and OMBE by four, enhancing thermodynamic stability considerably. Their upper pressure limits are determined by the evaporation mean free paths for OMBE, the deposition rate of ablated materials for PLD and the availability of RHEED for GOALL-Epitaxy. Regarding lower temperature limits, while PLD provides higher kinetic energy to deposited materials by laser ablation compared with OMBE at lower evaporation temperatures, GOALL-Epitaxy provides higher kinetics by laser ablation within single-atomic-layer ranges during growth, which is more flexible for lower temperatures compared with single-UC ranges for PLD. At higher temperatures, both GOALL-Epitaxy and OMBE are mainly limited by the substrate heater capacity (laser heater versus typical resistive radiation), while PLD also considers heat dissipation at higher pressures. The distinct growth parameter regimes for nickelates (represented by ReNiO_3_, where Re = rare earth), infinite-layer cuprates and finite-layer cuprates (e.g. La_2_CuO_4_ and YBa_2_Cu_3_O_7–δ_) involve both thermodynamic and kinetic considerations. The near-vertical boundary at lower temperatures represents a kinetic limitation: for instance, more complex and larger UC in finite-layer cuprates (e.g. YBa_2_Cu_3_O_7–δ_) requires higher kinetic thresholds for lattice formation compared with infinite-layer cuprates. The boundary at higher temperatures is determined by thermodynamic constraints, with greater oxidation power providing increased thermodynamic stability at elevated growth temperatures [43].

Finite-layer cuprates can be grown more readily by using conventional PLD or OMBE, thanks to their broad overlapping parameter spaces. However, the overlapping parameter spaces for nickelates and infinite-layer cuprates are significantly narrower, which limits the opportunities for optimization and the exploration of new phases and structures considerably. In contrast, the expanded parameter space of GOALL-Epitaxy offers significant advantages for the design and discovery of new materials. The superior oxidation capabilities support higher growth temperatures, which in turn enhances crystallinity by higher growth kinetics within single atomic layers. Additionally, GOALL-Epitaxy achieves atomic-layer precision—on a par with OMBE and exceeding the UC precision in conventional PLD—thereby optimizing structural precision while preserving material versatility.

In conclusion, GOALL-Epitaxy not only amalgamates the strengths of both OMBE and PLD while overcoming their limitations, but also surpasses them substantially in oxidation power. It vastly expands the design scope of strongly correlated electron systems with tailored functionalities to previously uncharted parameter spaces, such as higher-*T*_C_ superconductors, highlighting a transformative impact on the epitaxy of complex oxide materials.

## METHODS

### Nickelate RP phase growth

Growth temperatures are typically set to between 550°C and 800°C, with ozone chamber pressures ranging from 1 × 10^−5^ to 0.1 mbar. LaO*_x_* and NiO*_x_* targets are alternatively ablated by using a KrF excimer laser (λ = 248 nm, pulse duration 25 ns) for the sequential growth of different atomic layers. Stoichiometry control for various RP phases, encompassing the required pulse numbers for the completion and the laser energy of each atomic layer, is initially calibrated by using LaNiO_3_ film synthesis and subsequently fine-tuned. During deposition, the typical laser fluence on the LaO*_x_* and NiO*_x_* targets was ∼1.4 J/cm^2^ at 2 Hz and ∼1.4–1.8 J/cm^2^ at 2 Hz, respectively. The typical number of laser pulses was ∼90 for each LaO layer and ∼100 for each NiO_2_ layer. In the case of Sr doping, a (La, Sr)O*_x_* target was used instead of LaO*_x_*, in which the Sr ratio is ∼0.2. All growth of thin films was monitored in real time by using 30-keV RHEED. Substrates mounted on a flag-type sample holder were heated by laser with the highest temperature exceeding 1100°C.

### Atomic hydrogen reduction

The grown (La, Sr)NiO_3_ films without any capping were *in situ* transferred under an ultra-high vacuum from the oxidation growth chamber into a dedicated reduction chamber. The atomic hydrogen was generated by using commercial hydrogen sources from Dr. Eberl MBE-Komponenten GmbH. For different levels of oxygen content, the reaction temperature ranged from 250°C to 300°C and the flux ranged from 0.5 to 3 × 10^15^ atoms/cm^2^s.

### Infinite-layer cuprate growth

The infinite-layer cuprate films were grown on an (001)-oriented SrTiO_3_ or 0.05% Nb-doped SrTiO_3_ or NdGaO_3_ single-crystal substrate with a KrF excimer laser (λ = 248 nm, pulse duration 25 ns). During deposition, the laser fluence on the ceramic SrO*_x_* targets was 1.2 J/cm^2^ at 3 Hz, and on the ceramic CuO*_x_* and CaO_x_ targets was 1.5 J/cm^2^ at 3 Hz. The oxygen partial pressure was set to 1–2 × 10^−2^ mbar and the substrate temperature was maintained at from 550°C to 750°C. The typical number of laser pulses was ∼160 for each CaO*_x_* layer, ∼50 for each SrO*_x_* layer and ∼60 for each CuO*_x_* layer in the growth of stoichiometric CaCuO_2_, SrCuO_2_ and Sr_0.5_Ca_0.5_CuO_2_. After deposition, the SrCuO_2_ and Sr_0.5_Ca_0.5_CuO_2_ films were annealed at ∼1 × 10^−7^ mbar under 520°C for 10 min and the CaCuO_2_ films were cooled down to room temperature at a rate of 10°C/min at growth oxygen partial pressure. All growth of thin films was monitored in real time by using 30-keV RHEED. Substrates mounted on a flag-type sample holder were heated by laser with highest temperature exceeding 1100°C.

### Target preparation

CaO*_x_*, SrO*_x_* and LaO*_x_* targets are reactive in ambient atmospheres, forming hydroxides upon contact with water, while LaO*_x_* additionally absorbs CO_2_ from the air. To mitigate these reactions, these targets were sintered in a furnace within a glovebox under a dry Ar atmosphere. They were then mounted onto the target holders and rapidly transferred to the vacuum chamber through a load-lock system to minimize their exposure to air.

### Substrate preparation

To achieve sharp step and terrace surfaces on the TiO_2_-terminated SrTiO_3_ (001) substrates (Shinkosha, Japan), annealing processes were executed at 1100°C for a protracted period of 6 hours within an air atmosphere. For the LaAlO_3_ (001) substrates (MTI, China), an initial pretreatment entailed immersion in boiling deionized water for 15 minutes. Subsequent annealing was performed under identical temperature, time and atmospheric conditions to those for SrTiO_3_. The substrates were subjected to a repeated deionized water treatment-annealing protocol when needed, effectively yielding AlO_2_-terminated LaAlO_3_ substrates.

### XRD

Crystallographic characterization of thin-film specimens was performed by using SmartLab—an automated multipurpose X-ray diffractometer, from Rigaku Corporation, encompassing theta-2theta scans and reciprocal space mappings.

### STEM

STEM HAADF imaging of La_3_Ni_2_O_7_ and CaCuO_2_ was photographed by using a FEI Titan Themis G2 at 300 kV with a double spherical-aberration corrector and a high-brightness field-emission gun with a monochromator installed onto this microscope. The inner and outer collection angles for the STEM images (β1 and β2) were 48 and 200 mrad, respectively, with a semi-convergence angle of 25 mrad. The beam current was ∼80 pA for high-angle annular dark-field imaging and the EDS chemical analyses. All imaging was performed at room temperature. The cross-section STEM specimens of La_3_Ni_2_O_7_ and CaCuO_2_ were prepared by using a FEI Helios 600i dual-beam FIB/SEM machine. Before extraction and thinning, electron beam-deposited platinum and ion beam-deposited carbon was used to protect the sample surface from ion beam damage. The cross-section STEM specimen of the 1212 structure was prepared by using a Thermo Scientific Helios G4 HX machine and was protected by electron beam-deposited platinum and ion beam-deposited carbon before extraction and thinning. The STEM annular bright field and HAADF imaging of the 1212 structure was photographed by using a Thermo Scientific Themis Z at 200 kV with a spherical-aberration corrector. The EDS data of the 1212 structure were obtained by using the Super X FEI System in STEM mode.

### Low-temperature transport measurements

Electric transport measurements were performed in a closed-cycle helium-free system (base temperature of <1.5 K). The four terminal electrical measurements were carried out through either the standard lock-in technique with an AC current of 1 μA (13.333 Hz) or a Keithley 6221 current source and 2182A voltmeter in a delta-mode configuration.

### COMSOL simulation of ozone gas flow in the chamber

A multiphysics numerical simulation that integrated fluid flow and heat conduction was conducted by using a coupled approach. The finite element method was employed to solve the steady-state equations of fluid dynamics and heat transfer. The geometric model of the simulation was designed to closely mimic the experimental growth apparatus, with dimensions of 400 mm for both the diameter and the height of the chamber, and a 70-mm gap between the PLD target and the heating stage. The computational domain was discretized by using a free tetrahedral mesh. The simulation accounted for the properties of oxygen, specifying the density, dynamic viscosity and thermal conductivity accordingly. The temperature of the heating stage was maintained at 600°C, while the chamber walls were kept at 20°C. For the boundary conditions, the inlet was given a normal inflow velocity of 3 mm/s, while the outlet was treated as a pressure outlet and set to 1 Pa.

## Supplementary Material

nwae429_Supplemental_Files
