# Supplementary material for: Gigantic-oxidative atomic-layer-by-layer epitaxy for artificially designed complex oxides
Source: Natl Sci Rev. 2024 Nov 27;12(4):nwae429. doi: 10.1093/nsr/nwae429 (PMC11960094; doi:10.1093/nsr/nwae429)
Supplement: nwae429_Supplemental_Files [file nwae429_supplemental_files.zip › GOALL-Epitaxy SI_Final.pdf]

Supplementary Information

Gigantic-oxidative atomic-layer-by-layer epitaxy for artificially designed complex oxides

Guangdi Zhou[1#], Haoliang Huang[1,2#], Fengzhe Wang[1], Heng Wang[1], Qishuo Yang[1], Zihao Nie[1], Wei Lv[1], Cui Ding[2], Yueying Li[1], Jiayi Lin[1,3], Changming Yue[1], Danfeng Li[2,4], Yujie Sun[1,2], Junhao Lin[1,2], Guang-Ming Zhang[5,6], Qi-Kun Xue[1,2]*, Zhuoyu Chen[1,2]*

[1]Department of Physics and Guangdong Basic Research Center of Excellence for Quantum Science, Southern University of Science and Technology, Shenzhen 518055, China
[2]Quantum Science Center of Guangdong-Hong Kong-Macao Greater Bay Area, Shenzhen 518045, China
[3]Department of Physics, South China University of Technology, Guangzhou 510006, China
[4]Department of Physics, City University of Hong Kong, Kowloon, Hong Kong
[5]State Key Laboratory of Low-Dimensional Quantum Physics, Department of Physics, Tsinghua University, Beijing 100084, China
[6]Frontier Science Center for Quantum Information, Beijing 100084, China

[#]These authors contributed equally.
*E-mail: xueqk@sustech.edu.cn, chenzhuoyu@sustech.edu.cn

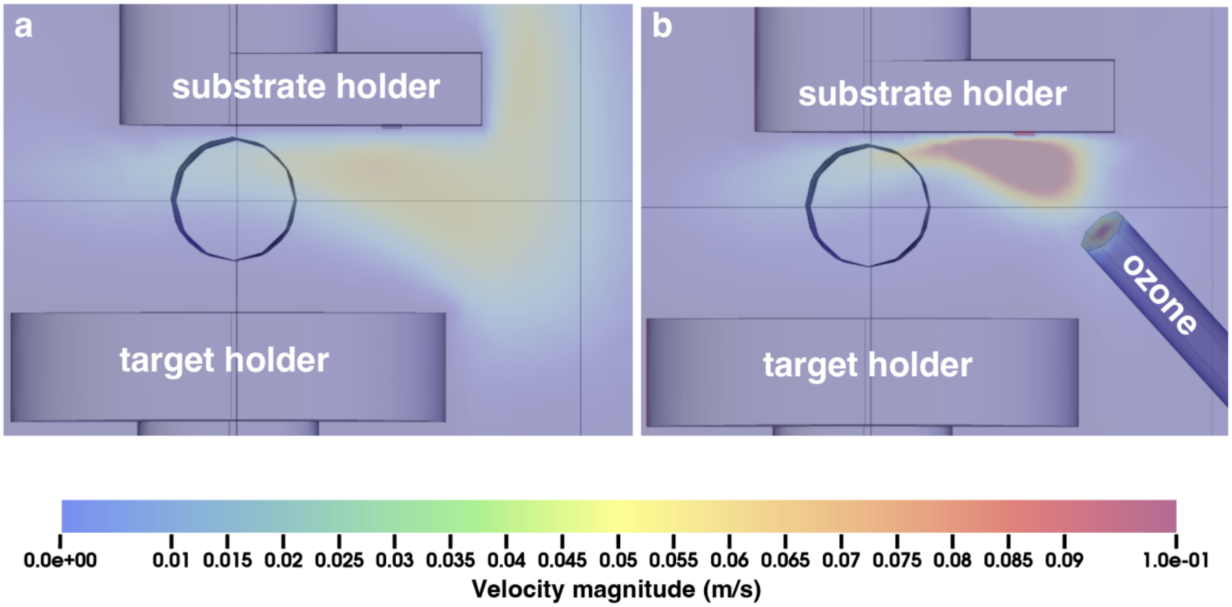

Fig. S1 | COMSOL simulation of ozone gas flow in the chamber. a and **b** depict the velocity magnitudes of ozone gas flow, with the nozzle opening situated 29 cm and 3.8 cm from the substrate, respectively. The velocity magnitudes at the substrate surface are 0.08 m/s for case a and 0.02 m/s for case b, indicating that positioning the nozzle closer to the substrate enhances the oxidation power by nearly half an order of magnitude. It is important to note that the oxidation power is predominantly determined by the initial contact of ozone molecules with the substrate due to their propensity for decomposition; therefore, we utilize velocity magnitude as a proxy for oxidation power rather than chamber pressure.

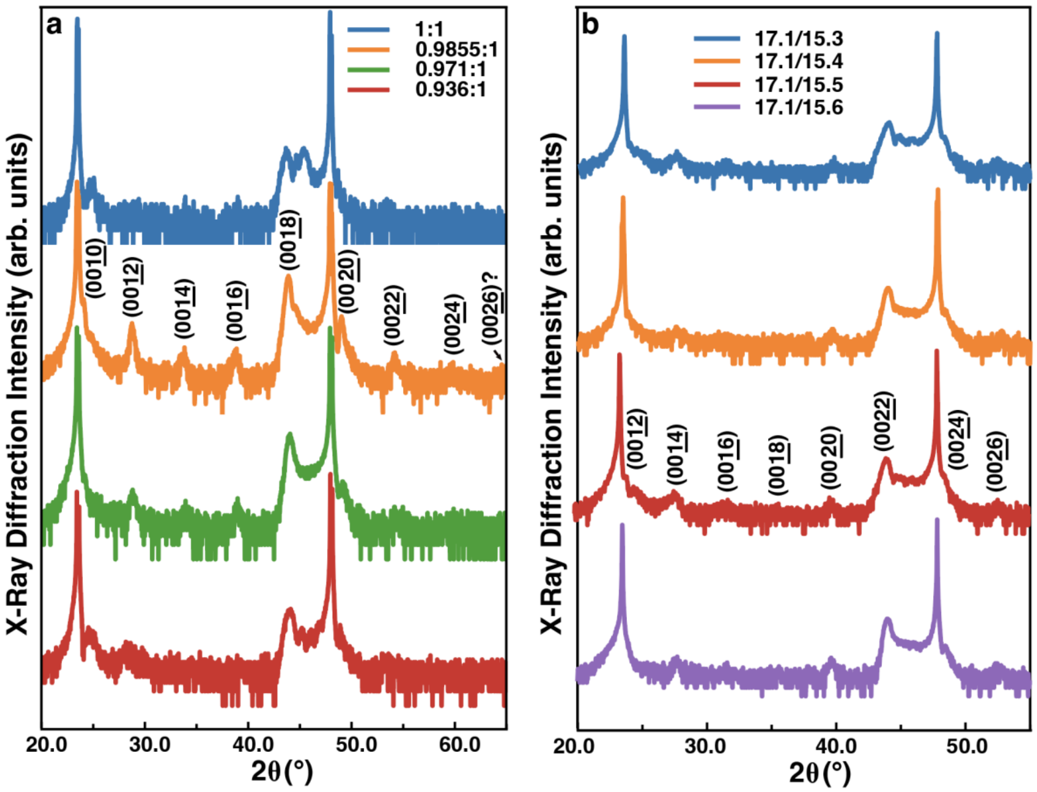

Fig. S2 | Stoichiometry control. In GOALL-Epitaxy, the stoichiometry can be tuned by varying the pulse number for each atomic layer (demonstrated in **a** for $La_5Ni_4O_{13}$), with the precision limit defined by single pulse of ablation, and by finer adjustment of the laser power (demonstrated in **b** for $La_6Ni_5O_{16}$). Different spectra in **a** represent different pulse number ratio between LaO_x target and NiO_x target ablations. Different spectra in and **b** correspond to different average laser energy of one pulse measured before entering the chamber in unit of mJ for LaO_x/NiO_x targets. Thickness of the films are around 10 nm. The theoretical stoichiometry precision limit, estimated based on the lowest adjustable digital increment of laser energy, is about 0.1%. In experiment, the smallest stoichiometry change is at 1%-2% level in **a**, and about 0.3% in **b**.

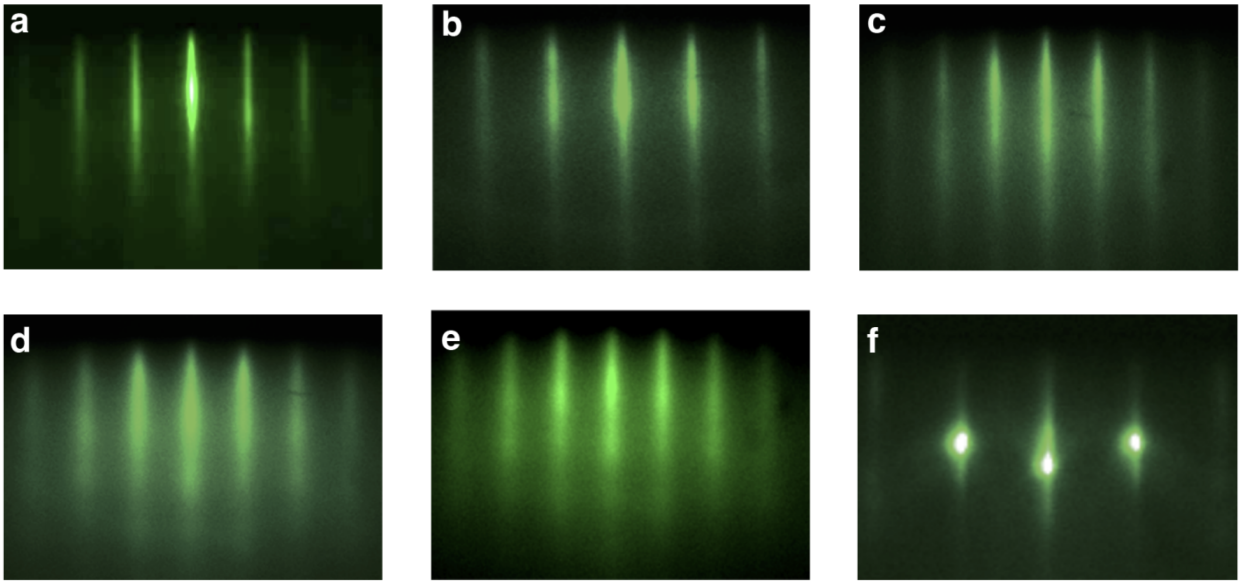

Fig. S3 | Reflective high-energy electron diffraction patterns of nickelate Ruddlesden-Popper phases after growth. a-**e**, one-layer to five-layer stacking structures $La_{n+1}Ni_nO_{3n+1}$, where n is the number of consecutive layers in one stacking block. **f**, infinite-layer stacking structure $LaNiO_3$.

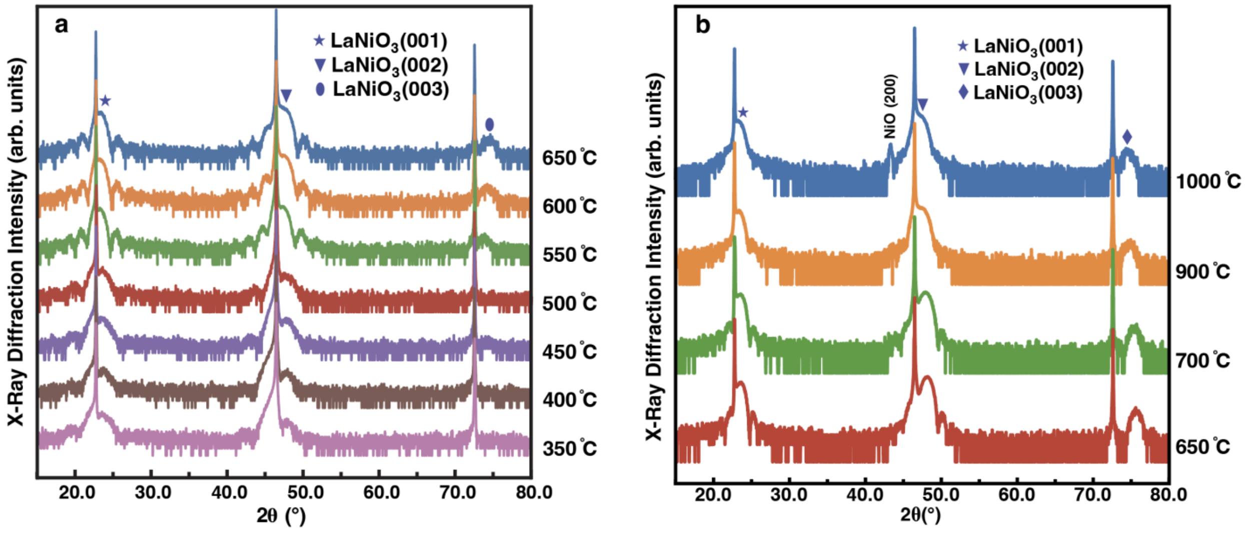

Fig. S4 | Expansion of growth temperature range for $LaNiO_3$ on $SrTiO_3$ substrate. GOALL-Epitaxy harnesses the kinetic energy of plasma from laser ablation for the top atomic layer being deposited, which enables the growth of single-crystalline epitaxial $LaNiO_3$ thin films at temperatures as low as 350 °C at 2×10^{-5} mbar O_3 chamber pressure (**a**) and as high as 900 °C at 0.08 mbar O_3 chamber pressure (**b**). At 900 °C, $LaNiO_3$ remains marginally stable, but at 1000 °C, the emergence of a NiO peak suggests thermodynamic instability. GOALL-Epitaxy extends the lower growth temperature limit, signifying higher growth kinetics, and enhances the upper growth temperature, indicating improved thermodynamic stability, compared to prior methods.

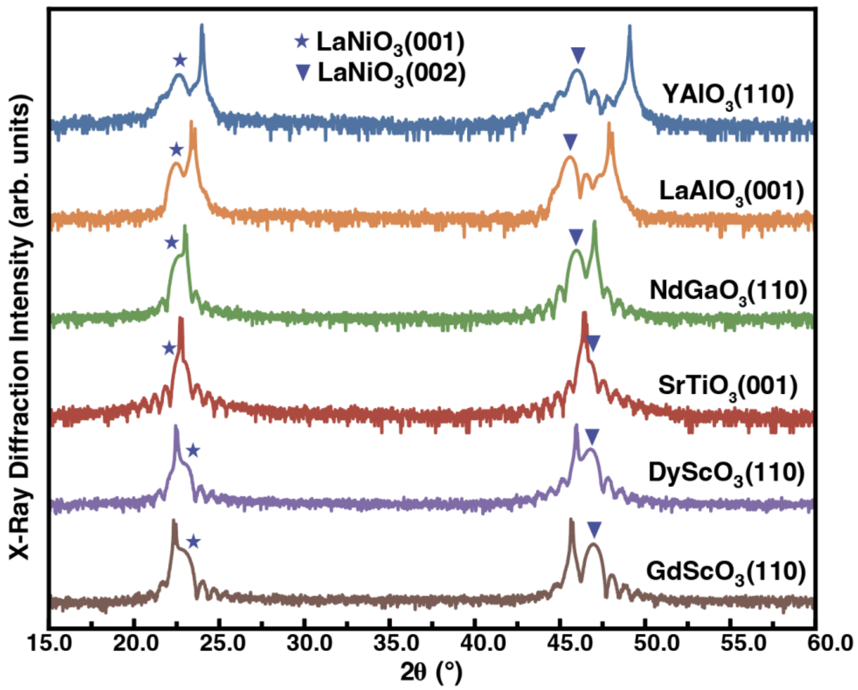

Fig. S5 | Wide-range epitaxial strain tuning of $LaNiO_3$. The quality of the films produced by GOALL-Epitaxy allows for a broad range of epitaxial strain across various substrates. The consistent shift in the out-of-plane lattice constant across films up to about 14 nm thick (grown at 650 °C and 2×10^{-5} mbar O_3 chamber pressure), from $GdScO_3$ to $LaAlO_3$ substrates, demonstrates the coherent application of strain, amounting to approximately a 3% change in the in-plane lattice constant. Notably, on $YAlO_3$ substrates, the out-of-plane lattice constant is smaller than that on $LaAlO_3$, despite $YAlO_3$ having a smaller in-plane lattice constant, suggesting a relaxation of strain under the extreme high compressive conditions of $YAlO_3$.

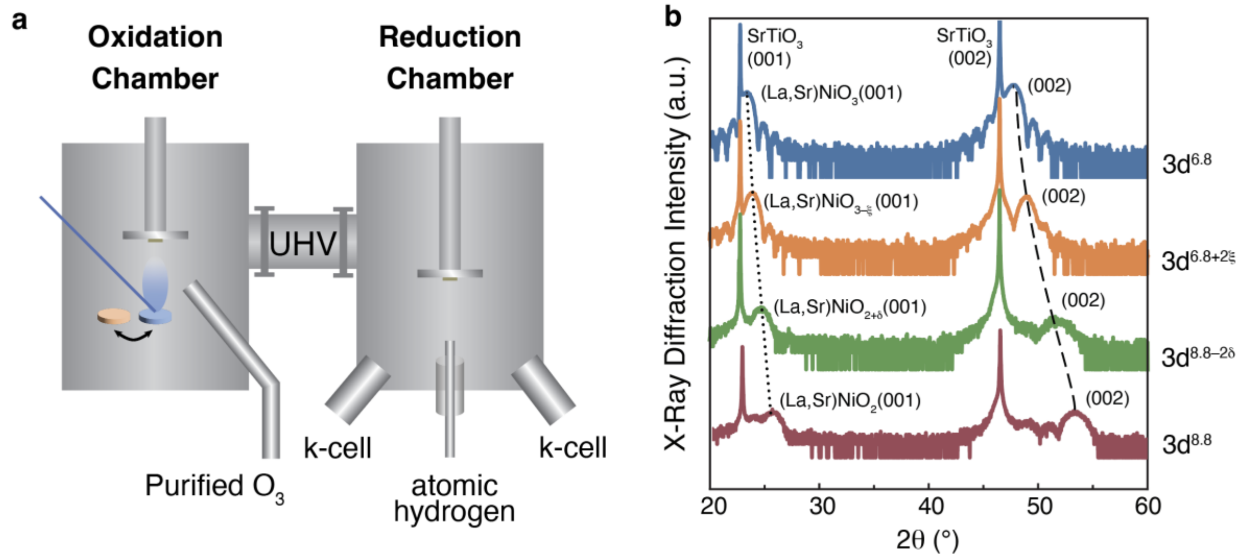

Fig. S6 | Continuous valence tuning via *in situ* atomic hydrogen reduction. a, Schematic diagram of the oxidation and reduction setup. K-cell: Knudsen effusion cell. UHV: ultrahigh vacuum. **b**, XRD data of a series of nickelates grown on $SrTiO_3$ substrate, having coherent infinite-layer structure but with different 3*d*-obital occupancy, tuned by varied oxygen content.

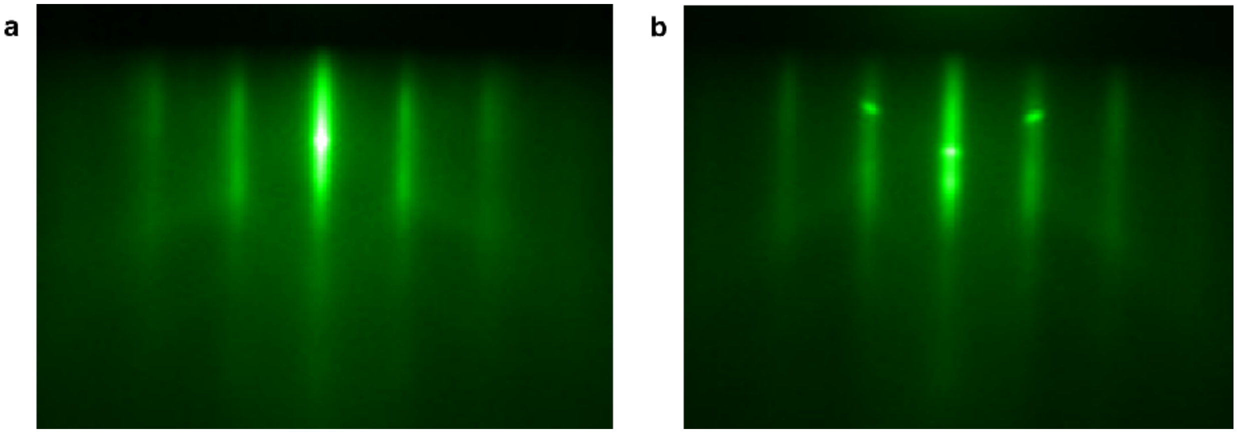

Fig. S7 | RHEED patterns of cuprates after growth. **a**, 20 unit-cell $SrCuO_2$ film on $SrTiO_3$ substrate. **b**, 20 unit-cell $CaCuO_2$ on $NdGaO_3$ substrate with 4 unit-cell $SrCuO_2$ buffer.

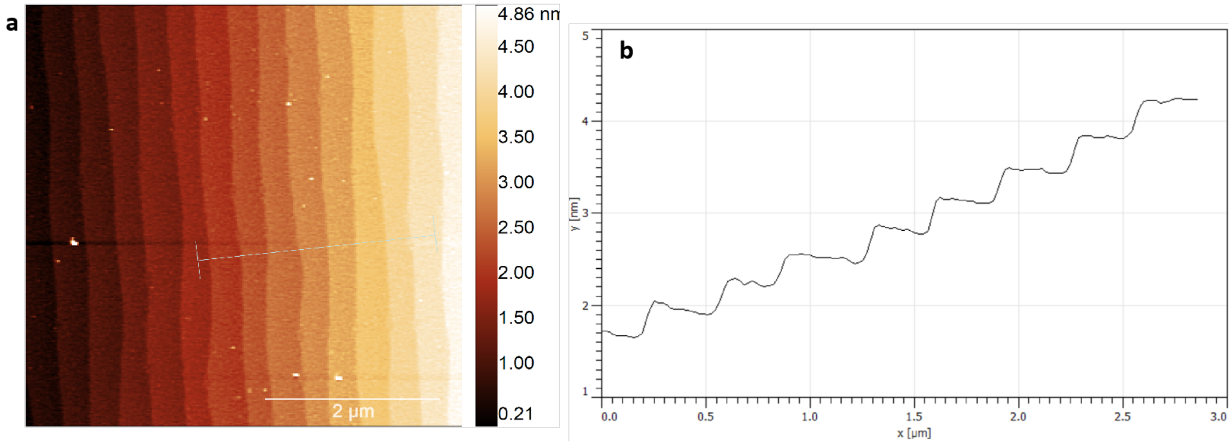

Fig. S8 | AFM topographic image of the $CaCuO_2$ film. a, Atomically flat surface of 15 unit-cell $CaCuO_2$ film on $NdGaO_3$ substrate with 4 unit-cell $SrCuO_2$ buffer. **b**, The atomic steps of $CaCuO_2$ film surface.
